# Endogenous neural stem cells characterization using omics approaches: Current knowledge in health and disease

**DOI:** 10.3389/fncel.2023.1125785

**Published:** 2023-04-05

**Authors:** Valentina Murtaj, Erica Butti, Gianvito Martino, Paola Panina-Bordignon

**Affiliations:** ^1^Division of Neuroscience, San Raffaele Vita-Salute University, Milan, Italy; ^2^Neuroimmunology, Division of Neuroscience, Institute of Experimental Neurology, IRCCS Ospedale San Raffaele, Milan, Italy

**Keywords:** neural stem cells, SVZ (subventricular zone), omics, neurodegeneration, transciptome analysis

## Abstract

Neural stem cells (NSCs), an invaluable source of neuronal and glial progeny, have been widely interrogated in the last twenty years, mainly to understand their therapeutic potential. Most of the studies were performed with cells derived from pluripotent stem cells of either rodents or humans, and have mainly focused on their potential in regenerative medicine. High-throughput omics technologies, such as transcriptomics, epigenetics, proteomics, and metabolomics, which exploded in the past decade, represent a powerful tool to investigate the molecular mechanisms characterizing the heterogeneity of endogenous NSCs. The transition from bulk studies to single cell approaches brought significant insights by revealing complex system phenotypes, from the molecular to the organism level. Here, we will discuss the current literature that has been greatly enriched in the “omics era”, successfully exploring the nature and function of endogenous NSCs and the process of neurogenesis. Overall, the information obtained from omics studies of endogenous NSCs provides a sharper picture of NSCs function during neurodevelopment in healthy and in perturbed environments.

## 1. Introduction

Endogenous neural stem cells (eNSCs), an essential source of cycling precursors in the adult brain, reside in the subventricular zone (SVZ) along the lateral ventricle, and in the subgranular zone (SGZ) of the dentate gyrus (DG) of the hippocampus (Luskin, 1993; Gonzalez-Perez et al., 2011). These stem cell niches are surrounded by peculiar microenvironments releasing factors that promote the maintenance and the differentiation of neural stem cells (Jin and Galvan, 2007).

The SVZ is composed of a heterogeneous group of cells, the type B1 radial glia cells (RG), expressing GFAP, GLAST, and GLT-1 markers, which generate the transit amplifying cells, intermediate progenitors, the type C (Blackmore and Rietze, 2013). The latter type generates the neuroblasts (type A cells, neuroblasts/NBs) after three to four symmetrical divisions, which migrate to the olfactory bulbs (OB) through the rostral migratory stream (RMS), where the new-born neuronal progeny arise (Ayala et al., 2007). Moreover, B1 cells are in contact with the ependymal cells that extend their end-feet through the ependymal layer to contact the ventricle and the cerebrospinal fluid (CSF). On the basolateral side of the ventricle, these cells contact blood vessels, where an exchange of soluble factors with the endothelial cells occurs (Shen et al., 2008).

In the SGZ, two types of neural progenitors, the type 1 and type 2 cells, reside. These cells generate new excitatory neurons that migrate at a short distance from the niche reaching the granule cell layer of the DG, where they integrate into the hippocampal circuits (Zhao et al., 2008). The heterogeneity in the origin and the generation of specific newborn neurons produced by those two cell types has been described (Chaker et al., 2016). Indeed, type B1 cells generate different subsets of OB interneurons based on their location along the lateral ventricle, anterior-posterior and dorsal-ventral axes, along with the expression of several important genes, such as Pax6 and Sonic hedgehog (Shh; Obernier and Alvarez-Buylla, 2019).

An additional peculiarity of the NSCs in both niches regards their ability to transit through quiescence, activation, and differentiation states. Moreover, NSCs show different degrees of quiescence and diverse ability to originate neurons and glial cells. The quiescent NSCs (qNSCs) are characterized by the expression of Sox9, Id2, and Id3 and the lack of proliferation markers; while the activated NSCs (aNSCs) express Egfr, Ascl1, Dlx1, and Dlx2 (Codega et al., 2014; Obernier and Alvarez-Buylla, 2019). Gene expression programs involving cell signaling, cell-cell communication, cell adhesion, and extracellular matrix (ECM) signaling define qNSCs, while transcription, translation, and DNA repair genes are typical of aNSCs (Codega et al., 2014).

Considering the structural difference between the two neurogenic niches, it is important to note that VZ-SVZ is involved in cortical development during early developmental processes. Corticogenesis involved the establishment of the olfactory bulb (OB) and the sixth-layer neurons, spatially generate from the deepest to the superficial layer of the cortical region (Ventura and Goldman, 2007). Hippocampal neurogenesis of SGZ, on the other hand, is involved in the generation of DG structure with the peculiarity to be active also in adult life, both in rodents and in humans (Sugiyama et al., 2013). These two niches are exposed to different signals. Neurogenesis, maintenance, and regulation of NSCs, and the transition from quiescence to activated states are all tightly regulated by extrinsic secreted factors provided by the niche and the ECM but also by intrinsic mechanisms (Ding et al., 2020).

Furthermore, NSCs are regulated by several neurotransmitters such as serotonin, dopamine, glutamate, GABA, acetylcholine, and noradrenaline (Berg et al., 2013). In particular, SVZ-NSCs receive GABAergic input from NBs, which inhibits their proliferation, while the SGZ-NSCs receive GABAergic input from local parvalbumin interneurons regulating hippocampal neurogenesis (Fernando et al., 2011; Song et al., 2012).

The maintenance and the quiescence of NSCs are controlled by chromatin regulators that affect chromatin accessibility, by repressor complexes and transcription factors that oversee transcriptional activity, and by cell cycle regulators (Andersen et al., 2014; Chen and Dent, 2014).

NSCs exert neurogenic and non-neurogenic functions both in physiological and pathological conditions, but their role is only partially characterized (Butti et al., 2014). Thus, a systematic investigation of the cell type specific roles and functions, and the characterization of the eNSCs regulators are needed to define a comprehensive landscape of NSCs. The novel omics-based approaches, with the availability of several high-throughput techniques such as genomics, epigenetics, transcriptomics, proteomics, and metabolomics, have allowed the study of fundamental regulatory mechanisms of endogenous NSC functions.

This review aims to recapitulate the results of high-throughput omics studies performed in physiological and pathological conditions, which have been conducted in the years (2017–2022). The focus will be on studies of genome variation, methylation profiling, non-coding RNA profiling, and proteins/metabolites profiling by high-throughput sequencing approaches. An overview of several omics techniques applied to eNSCs studies will be presented. Finally, multi-omics studies will be discussed, as the integration of different techniques is essential to completely unveil the biological systems under investigation.

## 2. Omics technologies applied to NSCs

Most studies on NSC profiling include transcriptome analysis, which consists of bulk and single cell RNA sequencing. This latter technique has enabled us to deeply dissect cell specific signatures and provide an overview of each cell phenotype. The final products of a specific phenotype can be defined by proteomics, large-scale studies of proteins, their structures, functions, and interactions within a biological system. Among the various proteomics techniques, matrix-assisted laser desorption ionization-time-MS (MALDI-MS) has been extensively performed, although antibody arrays are still widely used (Schumacher-Schuh et al., 2022). Metabolomics allows to study the intermediates and the end-products of metabolic processes that occur within a biological system, providing insights into the underlying biological processes. Metabolomics typically involves the use of analytical techniques such as mass spectrometry coupled with gas chromatography (MS-GC), and liquid chromatography (LC). Limitations of proteomics methodologies include limited dynamic range, as low abundance proteins may be missed or not detected, and the difficulty to detect post-translational modifications. There are also several limitations to the technology and methodologies used in metabolomics, as the coverage of metabolites in a sample can be limited by the sensitivity and selectivity of the analytical techniques (Kassem et al., 2021).

Chromatin status orchestrates NSC tasks from the early developmental to the adult stage, with changes in structural conformation and dynamics, DNA modifications, transcription factor binding, and histone activity (Lessard et al., 2007). Several methodologies have been developed to study NSCs at the epigenome level including Chromatin immunoprecipitation sequencing (Chip-seq), methylome analysis, and bulk/single cell Assay for Transposase-Accessible Chromatin (ATAC-seq; Zarnegar et al., 2017; Banerjee et al., 2019). The Chip-seq technique combines chromatin immunoprecipitation and next-generation sequencing to assess DNA targets for DNA-associated proteins and histone modifications (Nakato and Sakata, 2021). ATAC-seq, on the other hand, is used to assess chromatin open architecture, as it is based on the transposase enzyme activity which can map accessible chromatin regions (Yan et al., 2020).

Multi-omics approaches are defined as the integration of two or more omics techniques to highlight the complexity of the biological system under investigation (Hasin et al., 2017). Analysis of spatial distribution combined with histopathology, Magnetic Resonance Imaging (MRI), and metabolic maps for each cell type are essential to validate and confirm data derived from different omics approaches. Indeed, spatial transcriptomics represents a recent innovative technique that combines data from RNA transcriptome analysis and the spatial context of the specific tissue under investigation. It is often combined with scRNA-seq as *per se* spatial transcriptomic is still unable to capture single-cell resolution (Moses and Pachter, 2022). Nevertheless, spatial transcriptomic as well as other techniques combinations in a multi-omics approach are necessary to unveil and describe the precise mechanisms of neurodevelopment, especially as neurodevelopment highly depends on spatial evolution (Brancati et al., 2020).

## 3. Profiling of eNSCs in the physiological environment

### 3.1. Early events: from stemness and proliferation to cell fate decision and differentiation

The quiescent state of NSCs is characterized by the ability to retain mitotic capacity while remaining in a dormant state to preserve the stem cell niche during adulthood, while aNSCs proliferate and progress towards a differentiated progeny (Mohammad et al., 2019). The transition from a quiescent to an activated state has been thoroughly investigated by transcriptomics. An SVZ bulk-seq study reports several genes and transcription factors (TFs) that are commonly expressed by the two populations, while 433 genes are positively associated only with qNSCs, and 563 genes only with aNSCs, indicating substantial differences in the two populations (Morizur et al., 2018). Expression of the TF Klf9 was associated with the quiescent state, while Sdc1, encoding a member of transmembrane heparin sulfate proteoglycans with a major role in cell-matrix interactions and proliferation of neuronal precursors, was highly expressed by aNSCs (Morizur et al., 2018). In comparison, the SGZ is characterized by a high expression of Id4 TF which controls the quiescent state (Niola et al., 2012; Blomfield et al., 2019). As hippocampal stem cells remain mainly in the quiescent state, the description of the mechanism by which they remain dormant or get activated could guide the design of cell replacement therapeutic strategies for neurodegenerative diseases (Sorrells et al., 2018). In the Notch2 conditional KO, Id4 expression was significantly reduced, while forced Id4 expression induced differentiation towards the astrocyte lineage. Thus, Notch2 acts to maintain quiescence and includes Id4 as the main downstream regulator together with Hes1/Hes5 expression induction, which then represses the pro-neural TF Ascl1 and maintains NSCs in the dormant state (Zhang et al., 2019).

Both maintenances of a proliferative state and neurogenesis involve the expression of imprinted genes, expressed only by one parental allele, while the other is repressed (Ferguson-Smith, 2011). Igf2 parental gene expression is associated with NSC proliferation in the SGZ, while Dlk1 mono-allelic expression is related to postnatal neurogenesis enhancement in the SVZ (Ferrón et al., 2011, 2015). Recently, by RNA-seq analysis, novel imprinted genes have been involved in stem cell state preservation, whose expression is regulated by the TET3 enzyme, a member of the Ten-Eleven Translocation (TET) family of epigenetic regulators directly implicated in mammalian DNA methylation (Wu and Zhang, 2017; Montalbán-Loro et al., 2019). Upregulation of Small Nuclear Ribonucleoprotein N (Snrpn) imprinted gene in TET3-deficient NSCs leads to decreased neurogenesis in the OB together with loss of stem cell state, indicating direct repression of Snrpn by TET3. This mechanism leads to the maintenance of the stem cell niche by acting on the cell’s self-renewal capacity.

The post-transcriptional mechanisms that regulate NSCs differentiation and stemness are still poorly characterized. Protein synthesis is a dynamic process during neurogenesis: as expected, qNSCs exhibit low protein synthesis, which increases upon cell activation and decreases again in mature neuronal populations (Baser et al., 2019). Transcriptome and translatome variations revealed few common features between the early and late NBs transition, while higher differences were found during the differentiation towards mature cell populations. Translational repression begins in the late stage of NBs and is maintained in the neuronal lineage. Ribosomal mRNA and TFs, such as Sox2 and Pax6, were found highly repressed in the switch state between stem cells and early NBs. Interestingly, mTORC1 seems to be responsible for those TFs repression, as its level was lower in early NBs and higher in late NBs, where it exerts its repression activity. Indeed, mTOR signaling pathways influence cell cycle regulation and proliferation (LiCausi and Hartman, 2018).

The current literature shows different chromatin accessibility in activated vs. quiescent primary NSCs (Maybury-Lewis et al., 2021). ATAC-seq analysis showed distinct chromatin dynamics across the two NSC states with 67% accessible shared sites, named as stable accessible chromatin. In the aNSCs, high accessibility was found close to the Olig2 gene, while high accessibility of the Fgf1 locus was observed enriched in qNSCs. The variation between the stable and dynamic chromatin accessibility resides in the transcription start site (TSS). Stable chromatin was found in the promoter regions, while dynamic chromatin was found in the distal intergenic and intronic regions, meaning that promoter regions are crucial for NSC shift between these two stages. Enrichment analysis supports this notion, being stable chromatin associated with pathways regulating proliferation, translation, RNA polymerase II assembly, and metabolism, while dynamic chromatin was enriched for pathways associated with neuronal specification (Maybury-Lewis et al., 2021).

During early embryogenesis, one of the most important mechanisms governing embryonic stem cells’ (ESCs) function is the repression of differentiation and promotion of pluripotent genes by changes in chromatin accessibility, histone modification, and activity of nucleosome modifiers such as Asf1a (Sharp et al., 2001). The latter was recently found to act as nucleosome disassembly during murine ESC differentiation, by combined RNA-Chip-seq analysis (Gao et al., 2018). Surprisingly, its activity is not directed toward silencing pluripotent genes, but it rather affects differentiation genes, as in its absence the whole transcription machinery is delayed. Moreover, Asf1a promotes neuronal differentiation. On the other hand, the chromatin remodeler Chd8 is a repressor of cell differentiation, which instead supports cell pluripotency *via* association with Sox2, thus regulating chromatin accessibility (Sood et al., 2020). By RNA-seq, Chip-seq, and ATAC-seq multi-omics analysis of both Chd8^+/−^/Chd8^−/−^ mouse ESCs and differentiated NSCs, it has been demonstrated that in ESCs Chd8 maintains the open chromatin of the pluripotent genes, while in NSCs it represses Sox2 motifs (Goodman and Bonni, 2019). This finding is particularly significant for understanding the pathogenesis of neurodevelopmental disorders, as Chd8 mutations have been associated with autism spectrum disorders (ASD).

A study published in 2018 has collected several whole genome transcriptome datasets across various cell types including NSCs from SVZ and choroid plexus, glial cells, and microglia: bioinformatic analysis characterized the signaling pathways involved in neurogenesis and their ligands pattern (Cahoy et al., 2008; Beckervordersandforth et al., 2010; Zamanian et al., 2012; Codega et al., 2014; Israelsson et al., 2014; Silva-Vargas et al., 2016; Azim et al., 2018). The entire work focused on secretome pathways, and differentially expressed genes (DEGs) were validated across different cell types. The results revealed a huge contribution of the niche to neurogenesis, in which common and cell-specific ligands were described. The analysis of the SVZ lateral wall indicated that several ligands belonged to the Notch2 signaling pathway, chemokine pathways, and the AKT pathway. This study should be considered as a starting point for further meta-analysis to obtain a distinct picture of NSCs heterogeneity and uniqueness, and potentially elucidate the secretome of the hippocampal niche.

The transition from a quiescent towards an activated phenotype is directed by the transmembrane protein Lrig1, reported as a stem cell regulator in the peripheral districts (Marqués-Torrejón et al., 2021). A bulk RNA-seq analysis interrogating the differences between quiescent and activated SVZ NSCs revealed Lrig1 as highly upregulated in aNSCs, which might act as a critical regulator to help transition from quiescent to activation. Indeed, Lrig1 protein levels increase during the switch from quiescent to activated state inducing rapid cell cycle re-entry (Marqués-Torrejón et al., 2021).

#### 3.1.1. The role of RNA binding proteins

RNA binding proteins (RNABP) represent critical controllers of development (Parra and Johnston, 2022). The RNABP Quaking 5 (Qki5) has been found to decrease in neuronal progenitors and shown to regulate the transcription of cell adhesion molecule genes *via* binding to the intronic RNA (Hayakawa-Yano et al., 2017). A transcriptomic analysis of the Qki5-silenced E14.5 mouse cortex revealed increased cell differentiation, with preferential neuronal rather than glial cell generation. Qki5 and Qki6 are specifically expressed at the stemness state and decrease upon neural commitment. The authors elucidated the Qki5 RNAPB role, showing that it interferes with cell-cell interactions rather than stemness maintenance of the neural cells. Indeed, Gene Ontology (GO) analysis revealed a massive de-regulation in several biological pathways upon Qki5 silencing. Upregulation of cell adhesion, actin filament, cell projection, and microtubule suggest an important role of this RNABP in the progenitor to neuron transition (Hayakawa-Yano and Yano, 2019).

RNABPs are also responsible for translational regulation *via* binding untranslated regions (UTR; 5’ and 3’) during neurodevelopment (DeBoer et al., 2013; Zhang et al., 2016). Indeed, Celf1 RNAPB, an upstream regulator of 5’ UTR-driven translation, regulates the translation of the Elavl4 RNABP acting on radial progenitors to produce a balanced neocortical region (Popovitchenko et al., 2020).

Preservation of the adult SVZ-NSCs has been shown to be regulated by the RNABP Ars2, a conserved protein that acts as a transcriptional regulator of the Sox2 TF, involved in stem cell maintenance (Andreu-Agullo et al., 2011). Ars2 has been evaluated longitudinally during embryonic to adult hippocampal differentiation by Chip-seq, indicating time-dependent distinct Ars2 activities (Yu et al., 2020). Ars2 induces expression of the Sox2 TF and acts in the permissive chromatin conformation. The other enriched peaks include developmental genes along with oligodendroglial genes expressed only during the phase that precedes differentiation. Thus, Ars2 is a critical DNA regulator involved in progeny differentiation.

#### 3.1.2. Environment shapes NCSs

Environmental stimuli are no less important transcriptional regulators than TFs in shaping the NSC niche toward proliferation and fate commitment (Chiu and Dawes, 2012). For example, the maternal environment influences the proliferation and differentiation proprieties of NSCs during the embryonic stage (Wang et al., 2021). Bulk RNA-seq has shown that high levels of maternal estradiol decrease the proliferation rate of hypothalamic NSCs, with the involvement of long non-coding RNAs (lncRNAs). Single-cell transcriptomic analyses will help to clarify which cell type/s influence the rate of neuronal generation.

Notably, an RNA-seq study indicates that signaling *via* reactive oxygen species (ROS) regulates the fate of DG NSCs (Adusumilli et al., 2021). Neurons can adopt specific defense mechanisms against oxidative stress, however ROS levels in the DG seem to be crucial for quiescent maintenance, compared to the SVZ. In fact, DG-NSCs retain a higher level of ROS compared to SVZ stem cells, which contribute to the expression of less committed neuronal subsets, while genes involved in cell cycle inhibition are enriched. This result suggests that critical ROS levels shape proliferation in DG early-stage quiescent NSCs. On the contrary, low ROS levels observed in the SVZ niche are likely to drive cells toward cell cycle and differentiation (Adusumilli et al., 2021).

ROS levels are also associated with accelerated neuroepithelial cell differentiation (Özsoy et al., 2021). Intriguingly, a novel model of enhanced neurodevelopment has been proposed, which takes place during neurulation, involving specialized “cannibalized” neuroepithelial cells that are able to “eat” erythroblasts *via* endocytosis from the yolk sac, eventually acquiring the hemoglobin precursor (heme) molecule. The proposed mechanism leads to accelerated neuroepithelial cell development in a spatially restricted fashion, due to the activation of mitochondria that supply massive production of ROS by the electron transport chain. The specific heme-enriched NSCs showed a transcriptome enriched in the expression of the Notch1 repressor Numb that boosts β-catenin pro-differentiation activity, accompanied by the stabilization of adherent junction (Hirabayashi et al., 2004). Altogether, the proposed model needs further validation to demonstrate the strength of the suggested energy supply to support the overall machinery, and to test this hypothesis under hypoxic or anemic conditions.

Overall, these findings emphasize that ROS levels present in a critical time and space regulate brain development.

Stem cell niche-dependent cues affect the behavior of NSCs (Silva-Vargas et al., 2016). The NSCs in the V-SVZ, which is close to the lateral ventricle choroid plexus (LVCP), the main source of CSF, are indeed influenced by the LVCP secretome, as confirmed by proteomic analysis with antibody arrays (Silva-Vargas et al., 2016). Transcriptome analysis of the whole LVCP confirmed that it plays a central role in integrating local and systemic cues to dynamically modulate the V-SVZ stem cell niche.

### 3.2. Pre- and post-natal neurodevelopment profiling

The mechanisms of pre- and postnatal differentiation will be discussed in this section, focusing on the regulation of cell fate commitment. Overall, most investigations on the prenatal stage reported information regarding SVZ, while SGZ was mainly studied during adult life as the main source of adult neurogenesis. Thus, prenatal SGZ biology remains a gap in knowledge.

#### 3.2.1. Prenatal development: focus on corticogenesis

Time-dependent specification and cortical layers distribution of newborn neurons derived from RG require strict orchestration of events (Gao et al., 2014). Single-cell transcriptomic analysis, enriched by direct measurement of specific cell-type transcripts in defined CNS areas, compared to bulk analysis, has brought insights into the understanding of the heterogeneity of the NSC niche during development, thus revealing the complex mechanisms required during neurogenesis to regulate neuronal specification. Notably, a large single-cell transcriptomic work has developed a time-related atlas describing the heterogeneity and specificity of NSCs starting from apical and basal embryonic stem cells towards the neuronal lineage (Yuzwa et al., 2017). By analyzing E11.5, E13.5, E15.5, and E17.5 mouse cortex, the authors describe the critical genes involved in the developmental process by trajectory analysis. Increased percentages of neuronal genes and decreased proliferative capacity during time have been reported. The authors also describe how RG progenitors enter a non-proliferative state during adult neurogenesis, recapitulating a transcriptional program similar to qNSCs. Levels of transcripts in this critical period do not always match protein levels, specifically between E13.5 (deep and superficial layer), E15.5 (superficial layers), and E17.5 (neuronal specification finished). RG progenitors exhibit high levels of several transcripts of both deep and superficial neuronal subtype specification from E13.5 and E15.5, while some of those transcripts are still detectable at later time points, indicating RG priming toward the arrangement of both layers. However, the specific repressor effector Pum2/4E-T determines which of those subtypes will be generated at each time point of murine development. In the absence of Pum2/4E-T, aberrant co-expression of deep layer transcripts in the superficial layer occurs, miss-regulating the whole critical machinery and confirming that RG is primed toward the generation of diverse neuronal types (Yang et al., 2014; Zahr et al., 2018).

As mentioned in the introduction, tight regulation characterizes neuronal and glial development, involving many players such as TFs, transcriptional coactivators/repressors, and gene expression in a time-dependent manner along with environmental cues (Ma, 2006). The transcriptional activators YAP and TAZ play a prominent role in regulating the cell proliferation rate during embryogenesis (Lavado et al., 2021). In the absence of YAP, impaired astrocyte proliferation was reported (Park et al., 2016). Double YAP and TAZ KO however demonstrates the necessary role of these two co-transcriptional activators in maintaining RG proliferative capacity, structural organization, and ependymal cell generation (Lavado et al., 2021).

Bone morphogenetic proteins (BMPs) are crucial during neurogenesis and brain development (Bond et al., 2012). Hence, their dynamic changes during differentiation direct neurogenesis as well as gliogenesis are responsible for the hippocampal stem cell niche maintenance. Combined RNA-Chip-ATAC-seq analysis revealed that BMP2 influence neuronal differentiation early (E11) in mouse development, while at a later time (E14) it impacts astrogenesis. BMP-induced differentiation involves the Smads TFs that interact with Sox11 to drive neuronal differentiation, and switch to interact with Sox8 to promote astrogenesis (Katada et al., 2021).

Among BMPs known antagonists, Gremlin1 (Grem1) has been studied to elucidate its role in cortical neurogenesis (Ichinose et al., 2021). Indeed, Grem1 can induce excitatory deep cortical layer neurons, inducing upregulation of a set of genes involved in glutamate secretion. Moreover, Grem1 is specifically expressed by neuronal committed immature precursor cells, thus, being a good candidate for specific neuronal differentiation program enhancement (Ichinose et al., 2021).

Glutamatergic neuron generation is driven also by the master transcriptional regulator Sox1, shown to be sufficient to induce neuronal generation and glial fate repression. Specifically, Sox1 overexpression induces upregulation of neural stem cell genes and growth factors leading toward glutamatergic progeny (Baumann et al., 2019).

The generation of newborn cortical neurons depends on the activity of the COMPASS family histone methyltransferase co-factor Ashl2 (Li et al., 2019). Indeed, by RNA-Chip-seq mapping of NSCs in absence of the histone-modifier Ashl2, NSCs exhibit abnormal progeny generation, indicating a crucial role in NSC number maintenance as well as NSC state. Ashl2 affects also the WNT signaling pathways, as upon Ashl2 conditional KO, WNT activators were reduced. Data confirmed by Chip-seq show that Ashl2 binds promoter regions of those activators. Notably, Ashl2-related genes were found enriched in schizophrenia, epilepsy, and autism spectrum disorders.

NSCs and neural progenitors coexist in the embryonic as well as neonatal and adult stages, hence unveiling the mechanism of differentiation since early stages might bring an enormous insight into the global characterization of neurogenesis (Codega et al., 2014). A study assessing global gene expression by RNA-seq has shown the role of the chromatin remodelers Chd5 at an early stage of development (Hwang et al., 2018). Chd5, expressed in the post-mitotic neurons, in the neural progenitors of E14.5 rat cortical region, and hippocampal progenitor cells, plays a critical role during neurogenesis (Egan et al., 2013; Vestin and Mills, 2013; Nitarska et al., 2016). Upon Chd5 depletion, NSCs differentiation was directed toward the astrocytic rather than the neuronal lineage. Boosting of astrocyte differentiation was due to translation disruption of Mash1, a main pro-neural TF that is essential during early differentiation (Hwang et al., 2018). These results suggest a strong Chd5 involvement in the cell fate decision in both cortical and hippocampal NSC niches at an early stage of differentiation.

#### 3.2.2. Prenatal development: V-SVZ embryonic profiling

The ganglionic eminence (GE) has been investigated to unveil positional diversification of NSCs in the lateral (LGE), medial (MGE), and caudal (CGE) ventricular regions. GE is the area where forebrain GABAergic neurons differentiate during the initial embryonic cell fate commitment (Lee et al., 2022). Many investigations focused on late/adult corticogenesis but there is a lack of knowledge regarding the heterogeneity and the transcriptional profile of embryonic forebrain including LGE, MGE, and CGE. In a scRNA-seq study, the analysis performed on E12.5 mice revealed distinct gene expression profiles in the cortex vs. ventricular regions and a great overlap between LGE and CGE. A GE atlas reports several clusters belonging to progenitor and post-mitotic cells in which Nkx2-1, Lhx6, and Sst genes were associated with MGE, while Ebf1 and Mapt were found in the LGE and CGE, which identify GABAergic projection neurons. A heterogeneous cluster expressing Npy and Sst transcripts was present in the LGE and CGE indicating migratory interneurons deriving from the MGE. The Igfbp5 gene transcript was observed to be exclusively expressed by CGE at E12.5 and could be used to direct future high-throughput studies of this specific population, unveiling the specific transcriptional profile of GE (Lee et al., 2022).

The differences between embryonic/adult GE and cortical NSCs have been analyzed based on the hypothesis that the two populations coexist and might share the same transcriptional profile (Borrett et al., 2020). The analysis covering the embryonic and the adult stage (E14; E17; P2; P6; P20; P34; P61) identified very few differences between the two populations, mainly associated with neuronal lineage specification and positioning. Overall, the environment shapes the activation, proliferation, and fate determination of future neurons also after birth. Some of the involved environmental factors have been identified, still, the switch program from excitatory toward inhibitory progeny directed by the environmental niche has not been fully elucidated.

Analysis of individual transcripts using RiboTag mouse models allows identifying the mRNA transcripts that are being actively translated in specific cell types. It has been shown that the protein product and the corresponding gene expression do not always correlate when the NSCs differentiate into neurons *in vivo* (Baser et al., 2019). Adult neural stem cells translate abundant transcripts with little discrimination, and post-transcriptional regulatory mechanisms mark the onset of differentiation (Baser et al., 2019). As an example, translational repression of mRNAs that encode the stem cell identity factors Sox2, Pax6, and components of the translation machinery, is a necessary condition to allow the differentiation of the neurogenic lineage.

#### 3.2.3. Non-coding RNAs profiling during neurogenesis

In the last years, lncRNAs, specific transcripts that do not encode proteins, have been shown to regulate important biological functions, including neurogenesis (Briggs et al., 2015; Duran et al., 2019). The lncRNA Pnky has been described to be crucial during corticogenesis by RNA-seq (Andersen et al., 2019). The Pnky gene is localized near the pro-neural TF Pou3f2, and although its deletion does not affect Pou3f2 expression, it leads to loss of proliferation and decreased cortical neurogenesis. Another lncRNA named RUS, located upstream of the transmembrane protein Slitrk3 is involved in neurite outgrowth by regulating *in cis* this nearest upstream gene (Aruga et al., 2003; Schneider et al., 2022). However, upon RUS silencing, not only Slitrk3 expression was found downregulated, but also Notch- and Shh-related genes were affected. Indeed, activation of Notch signaling leads to qNSC maintenance while inactivation of the Shh pathway results in lower NSC proliferation (Engler et al., 2018; Mase et al., 2021). Such results are in line with transcriptomic data that, upon RUS depletion, show reduced expression of proliferative markers as well as neuronal differentiation genes, while pro-apoptotic and cell death genes were found to increase. Altogether, these studies highlight that lncRNAs are crucial regulators of brain cell development (Schneider et al., 2022).

LncRNAs have been also assessed by applying a novel technique for chromatin conformation capture, named NG Capture-C, which allows to simultaneously reconstruct gene networks in multiple cell types, and to reveal how chromatin conformations shape gene activity (Davies et al., 2016, 2017). This technique has been specifically applied to the lncRNAs that are co-expressed with their near protein-coding genes, specifically the Paupar lncRNA-Pax6 gene pair (Pavlaki et al., 2022). Data revealed complex cis-regulatory interactions in both Paupar and Pax6 promoter regions related to their paired co-expression in the brain.

MicroRNAs (miRNAs) are also relevant for basal progenitor amplification and neurogenesis (Cremisi, 2013). Two miRNAs are of particular interest: miR137, involved in the enhancement of ECM molecule Cd63 along with downregulation of pro-neural gene Myt1l, thus leading to proliferation maintenance, and miR-122, which acts at a post-mitotic level in late neurogenesis reducing neuronal maturation. These two effectors are crucial for cortical layer I-II identity acquisition (Tomasello et al., 2022).

Besides miRNAs and lncRNAs, also circular RNAs have emerged to play a role during neuronal differentiation and brain development (Piwecka et al., 2017). Circular RNAs include transcripts that are covalently linked together 3’- 5’ in a circular manner thus making them more resistant to exonuclease actions compared to linear RNAs (Jeck et al., 2013). A study performed in proliferative NSCs during differentiation and in newborn neurons revealed several up- and down-regulated circular transcripts that were associated with synaptogenesis/neuronal differentiation and cell cycle respectively (Dori et al., 2019). The same investigators in 2020 published a complete catalog of miRNA transcripts expression during corticogenesis in several neural cell types (Dori et al., 2020). Lateral embryonic cortices were analyzed in a double reporter mouse for characterization of proliferative, differentiating, and newly formed neurons revealing 163 putative novel miRNAs sequences, actively involved in post-transcriptional modifications during brain development.

#### 3.2.4. Postnatal development: neurogenesis

A study based on RNA-seq analysis of postnatal neurogenesis investigated the regional specification of neuronal commitment and stem cell control systems (Tiveron et al., 2017). In the dorsal SVZ, Pax6, Tbr1/2, Emx1/2, and Ngn1/2 genes are key actors during corticogenesis, inducing predominantly GABAergic and dopaminergic neurons, while GABAergic neurons are induced in the lateral SVZ. The analysis of postnatal OB time-dependent neurogenesis unveiled the role of the zinc finger TFs Zic1 and Zic2 in the dorsal neuronal subtype commitment, which induces GABAergic interneurons differentiation by suppressing dopaminergic fate commitment. Zic1 and Zic2 were found evolutionary conserved, as also *C. elegans* represses the dopaminergic commitment by the same mechanism (Tiveron et al., 2017). Pax6, a gene involved in fate commitment, has also been shown to induce interneurons and dopaminergic phenotype exclusively in the presence of Meis2 TF during the pre-differentiation stage (Hau et al., 2021).

The heterogeneity of the NSCs is well known, however, whether the stem cell progenitors in the adult pool at different anatomical locations are transcriptionally similar remains under investigation, and different results lead to discordant hypotheses. Indeed, spatial localization drives neuronal specification during the early developmental stage (Delgado and Lim, 2015; Delgado et al., 2020). B cells have been profiled by scRNA-seq techniques identifying specific mRNA transcripts of the dorsal and ventral spatial organization (Cebrian-Silla et al., 2021). Urah, the gene for urate hydrolase, and Dio2 encoding the iodothyronine deiodinase define the dorsal domain, linking them to glial lineage specification, while Crym, encoding thiomorpholine-carboxylate dehydrogenase, defines the ventral territory, pushing different neuronal differentiation overlapping with the transcription factor Gsx2 found in dorsal clusters. The interesting point is the fact that all these genes are linked to the thyroid hormone signaling pathway, as other thyroid hormone-related genes were found upregulated (Rudqvist et al., 2012; Cebrian-Silla et al., 2021; Luongo et al., 2021). Altogether, these results indicate a specific molecular pathway linked to the dorsal SVZ region.

A multi-omics study investigated the generation of dopaminergic (DA) medium spiny neurons expressing DA receptors 1 and 2 (DRD1, DRD2) by the combination of bulk RNA and Chip-seq (Xu et al., 2018). In the LGE, Sp8, and Sp9 TFs are crucial for the differentiation of the DA progeny. Sp9 binds the Six3 promoter and other Six3-related enhancers, inducing DRD2 expression by directly regulating the Six3 gene, which controls cell proliferation and differentiation. Sp9 has been also associated with MGE-controlling cortical interneurons, as a 50% reduction of this progeny has been found in the absence of Sp9 (Liu et al., 2019).

An important aspect of neurogenesis is the NSC positional identity and maintenance of regional differences along the dorsoventral axis (Merkle et al., 2007). Ventral NSCs express Nkx2-1 that under Shh control induces ventral cell progeny (Delgado and Lim, 2015). Recently mixed-lineage leukemia 1 (Mll1) gene was also found to be part of the preservation of the Nkx2-1 positive cell subset (Delgado et al., 2020). Indeed, mRNA expression and chromatin analysis revealed that transient MIl1 inhibition induces NSC progeny shift to anterodorsal specification.

Single-cell transcriptomic analysis of the OB region, including cortical and V-SVZ NSCs, has allowed genetic fate mapping of neuronal progenitors toward mature OB neurons (Mizrak et al., 2020). A thorough representation of the OB newborn neuronal landscape revealed an important network of cellular crosstalk among NSCs, which is critical to maintaining homeostasis of the progeny and preventing tissue damage. In the adult V-SVZ stem cell niche that is constantly exposed to the WNT ligands from the CSF, the intermediate stem cell express Notum, a negative regulator of WNT, orchestrates normal neurogenesis by repressing the proliferative state of neighboring NSCs, thus maintaining a regular rate of proliferation and differentiation (Lehtinen et al., 2011; Mizrak et al., 2020).

Most recently, it has been shown that in the postnatal V-SVZ, two different NSCs pools co-exist (Baur et al., 2022). Specific adult basal NSCs were found, which seem to mainly contribute to the OB interneurons generation, rather than the already known apical progenitors. The two populations were transcriptionally profiled indicating that different microenvironments finely regulate the apical and basal NSCs post-natally, depending on the Notch signaling. Indeed, Notch signaling effectors Tlx/Nr2e1, along with Delta1 (Dll1) and Jagged1 (Jag1) ligands, and Hes1/Hes5 effectors were differentially expressed in the apical and basal quiescent, activated, and transitioning progenitors. Apical qNSC revealed lower expression of Notch-related genes, while basal qNSC was enriched with Jag1 expression. Basal aNSCs showed high expression of both ligands, while Jag1 was the only up-regulated in activated apical NSCs. Finally, in intermediate progenitors, Hes5 was higher in basal NSCs. Altogether, this data describes the heterogeneity of the postnatal SVZ niche showing a Notch signaling-dependent specific cell distribution (Baur et al., 2022). Notch signaling pathways inducing neurogenesis are also shaped by the expression of Foxp1 (Braccioli et al., 2017). Upon Foxp1 gene silencing, several up- and down-regulated genes involved in the Notch signaling pathway and neurogenesis, respectively, were detected. Indeed, Foxp1 absence induces cells toward oligodendrocyte (Ol) lineage specification, as higher myelin-related Mbp gene expression was found. Moreover, Foxp1 absence enriches the expression of Notch ligand Jag1, lowering neural differentiation (Braccioli et al., 2017).

Variants of the transcription factor Foxp1 have been associated with neurodevelopmental disorders by multi-omics analysis including RNA-seq and Chip-seq (Braccioli et al., 2017). Foxp1 has also been linked to differentiation and neurogenesis (Li et al., 2015). In fact, Chip-seq analysis revealed an association of the Foxp1 gene to Sox3 and Nr2f1/Tlx transcription factors suggesting possible interactions during neurogenesis. Moreover, Foxp1 acts as a Notch modulator by binding the Jag1 promoter, thus inducing Jag1 repression with a subsequent increase in differentiation. RNAseq data confirm the enrichment in neurogenesis, synaptic regulation, and Notch signaling pathways associated with Foxp1 (Braccioli et al., 2017).

Among neurodevelopmental disorders, intellectual disability, and autism spectrum disorders are the most common (Mefford et al., 2012). SET Domain Containing 5 (SETD5), a gene whose mutation has been linked to those disorders, has been functionally characterized in neural development (Sessa et al., 2019). SETD5 is a chromatin accessibility regulator that has been shown to promote transcription elongation and exhibit H3K36 lysine methyltransferase activity during neuronal development (Deliu et al., 2018).

A recent article that performed an RNA-seq analysis of the mouse SVZ demonstrated that NSCs express the insulin growth factor binding protein L1 (IGFBPL1) that can be used as a marker of adult NSCs, specifically of neuroblasts. This protein is involved in the insulin-like growth factor-1 (IGF1) signaling pathway, which regulates the proliferation, survival, and axonal growth of the striatal neurons. Indeed, mice with specific ablation of IGFBPL1-positive NSCs show morphological and functional alterations of medium spiny neurons, and cognitive impairment (Butti et al., 2022).

It is important to consider that, in the post-natal V-SVZ niche, the ependymal cells co-exist with qNSCs, RG progenitors, and NBs. Ependymal cells are ciliated structures that interact with ventricle fluid forming the vertical system, and may represent a dormant stem cell niche of endogenous NSCs (Quaresima et al., 2022). Transcriptional profiling from transgenic mice specifically targeting ependymal cells revealed a unique gene expression pattern of those cells despite they share some of the classical NSC transcriptional patterns (Shah et al., 2018). Ependymal cells display a specific set of genes related to cilia, which makes them a unique cell cluster separated from the stem cell pool, clearly identifiable by the single cell transcriptome analysis. Yet, further studies would support this notion and clarify the role of common NSCs genes in the ependymal cluster. Furthermore, clia-related genes were confirmed in a GO analysis of different published datasets at several developmental stages (MacDonald et al., 2021). This study established a solid role and evolutionarily conserved mechanism of ependymal cells in metal ion-related function, setting the basis for further investigation of the ependymal cell role in perturbed environments.

Adult neural stem cell niches, defined based on their neurogenic potential (subependymal and medial-ependymal zone- S/MEZ and OB) and non-neurogenic potential (cerebral cortex), have been profiled by proteomic analysis (Kjell et al., 2020). A first insight links SEZ and MEZ to similar proteome profiles as they well separate from cortical and OB specimens in the principal component analysis (PCA). However, many proteins (4,786) were found differentially abundant in the four regions analyzed. Specifically, SEZ was enriched for oxidative phosphorylation and gene regulatory proteins, while OB was enriched for proteoglycans key for cell migration during neurogenesis. The cortical region was, on the other hand, enriched for synapse-associated proteins. MEZ and SEZ differences reside in proteins related to neurogenesis, which were higher in SEZ, specifically Tenascin-c (Tnc), an extracellular matrix protein. Indeed, matrix-associated proteins (matrisome) provide crucial structural support to sustain signaling pathways (Naba et al., 2012). Moreover, tissue stiffness, another critical parameter in neurogenesis and neurite outgrowth, is more pronounced in the SEZ compared to the MEZ niche, suggesting a high abundance of basement membrane structures and associated ECM proteins in this specific region.

Finally, the mechanisms of long-term self-renewal and the potential for differentiation of NSCs in the adult brain are beginning to unveil thanks to multi-omics studies. A central role of topoisomerase IIa (Top2a) has been recently described by integrating RNA and Chip-seq of mouse adult brain (Qin et al., 2022). Top2a acts on the cell cycle regulation by increasing NSCs proliferation. The whole machinery includes direct binding of Top2a to the ubiquitin protein Usp37, which is shown to be its direct genomic target.

Besides gene expression, chromatin accessibility, and TFs activity, adult NSC maintenance and division are regulated also by mitochondrial dynamics and metabolic plasticity (Xie et al., 2016; Iwata et al., 2020). Combined *in vitro* NSC metabolomics and proteomics profiling reveals an important role of the protease Ymel1 in controlling mitochondrial NSC metabolism and proliferative rate (Wani et al., 2022). Indeed, premature neuronal differentiation accompanied by decreased proliferation occurred in Ymel1 absence, leading to dysregulated mitochondrial proteome dynamics.

#### 3.2.5. Postnatal development: gliogenesis

During brain development, non-neural cells—differentiated astrocytes and oligodendrocytes—are generated and play a crucial role during adult life in maintaining CNS homeostasis (Arai and Lo, 2017).

In the course of cortical neurogenesis, intermediate progenitor cells enriched in the expression of the Gsx2 TF were identified by scRNA-seq, which could not only generate OB interneurons but also promote glial cell differentiation (Zhang et al., 2020). The appearance of Gsx2-expressing progenitors with multipotent features indicates that gliogenesis occurs before the end of neurogenesis, as shown by the high expression of Ols markers in the Gsx2 positive cell subset upon induction of the Shh signaling cascade, which upregulated expression of the zinc finger Gli1 and the Shh receptor Ptch1, along with expression of markers of OB interneurons and cortical Ols lineage.

Gli3 and Shh orchestrate the switch between differentiation of neuronal and glial progenitors: when Shh is upregulated, Gli3 expression decreases in a specific population, driving the differentiation of Gsx2 positive intermediate progenitors toward the cortico-oligodendroglial lineage (Zhang et al., 2020). The presence of Olig1-Gsx2-expressing NSCs was also confirmed in another study showing that these cells represent the majority of NSCs in the postnatal brain, and indicating that different newborn neurons across various brain regions derive from heterogeneous NSCs populations residing in different SVZ sub-regions (Del Águila et al., 2022). These findings should be complemented by spatial single-cell transcriptomics approaches to complete an atlas of NSC differentiation with higher spatial and temporal resolution.

Sex differences and a wall-specific neurogenic fate commitment have been detected by scRNAseq study investigating the whole SVZ stem cell niche (Mizrak et al., 2019). While both lateral and septal walls are enriched in neurogenic potential, the septal wall shows a pronounced gliogenic fate, with a higher rate of oligodendrocyte precursors cells (OPCs) in males compared to female mice. A possible mechanism contributing to this difference could be the specific activation of the myelinating system under the influence of sex hormones. However, this speculation needs to be validated on specific demyelinating diseases such as multiple sclerosis (MS), that predominantly affects females (Swamydas et al., 2009; Sellner et al., 2011).

Bulk gene expression analysis of *in vitro* differentiated Ols revealed that the Sox4 TF inhibits Ols differentiation *via* expression of the Hes5 TF (Braccioli et al., 2018). GO indicated that CNS differentiation arises from the activation of the Notch signaling pathway, suggesting that Sox4 enhances neurogenesis and downregulates oligodendrogenesis towards mature and myelinating Ols. Indeed, Hes5 silencing induced Ols differentiation *in vitro*.

The involvement of specific lncRNA in cell fate determination of the oligodendroglial lineage has also been investigated by integrating RNA and Chip-seq (Wei et al., 2021). As reported in subchapter 2.2.3, lncRNAs play a fundamental role during brain development by binding to the chromatin modifiers and leading to changes in the chromatin landscape, thus affecting gene expression (Roberts et al., 2014). Notably, during Ols differentiation, the Olig2 gene regulates many lncRNAs acting as activators or repressors, as they exhibit OLIG2 biding sites (Wei et al., 2021).

The characterization of neural and non-neural cell subsets by computational analysis of RNA kinetics has unveiled the dynamics of changes in gene expression under developmental transition (Zywitza et al., 2018). The single-cell fate can be evaluated by calculating the ratio between spliced and unspliced mRNA, which defines RNA velocity as the time derivative of the gene expression state. This approach was applied to the single cell analysis of the mouse SVZ, showing that astrocytes and qNSCs exhibited stable gene expression, while great changes in the transcriptome occurred during the transition toward amplifying progenitors and returned stable when late NBs are produced (La Manno et al., 2018).

The balance between neurogenesis and oligodendrogenesis is strictly related to transcription regulators such as Dlx1/Dlx2 that promote neuronal progeny, and Olig1/Olig2 that induce Ols differentiation (Petryniak et al., 2007; Silbereis et al., 2014; Lindtner et al., 2019). In this regard, chromatin conformations are crucial in determining fate commitment through accessibility to regulatory element regions such as putative cis-regulatory elements (CRE). By applying ATAC-seq, a strong interaction between Dlx1 and Rbbp4/Rbbp7 NuRD chromatin repressive complex has been shown (Price et al., 2022). Notably, during SVZ cell differentiation, chromatin accessibility is reduced and mediated by Dlx1–Rbbp4/7 contact leading toward repression of Ols differentiation. On the contrary, in the absence of Dlx1, Ols differentiation prevailed. Thus, nNuRD chromatin remodelers are crucial at this step to decide whether to initiate neuronal or oligodendroglia commitment.

Astrocyte differentiation has been hypothesized to be influenced by the activation of NF-κB, a TF known to be involved in brain development (Kovács et al., 2004; Birck et al., 2021). Indeed, an inflammatory stimulus like TNF-α induced the maintenance of SVZ glial progenitors rather than promoting their differentiation toward astrocytes, as shown by RNA-Seq analysis. Future *in vivo* studies applying omics techniques will provide insights into the crucial role of NF-κB during astrocytic differentiation. Astrogenesis is also supported by proteins of the high-mobility group nucleosome-binding Family (Hmgn1, Hmgn2, Hmgn3; Nagao et al., 2014). Furthermore, RNA-ATAC-Chip-seq highlight specific TFs activity during astrocyte differentiation in the mouse brain, which involves Nfia and Atf3, while Runx2 drives their maturation (Tiwari et al., 2018).

**Figure 1:**
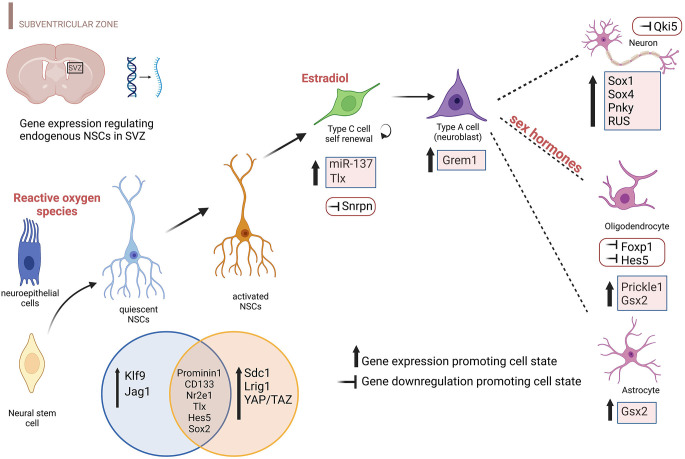
Environmental factors and differentially expressed genes promoting specific NSC states in the SVZ. The cartoon highlights the transcript perturbations that favor each cellular state during brain development including the prenatal and postnatal period, specifically for the SVZ. Both upregulation and downregulation of gene expression are reported including environmental factors that influence neuroepithelial cells (ROS), NBs (estradiol), and Ols (sex hormones). Genes reported include protein-coding genes, TFs, lncRNAs, and microRNAs. This figure has been realized using BioRender.

Gene expression perturbations and environments shaping different NSCs subtypes in the adult SVZ are depicted in Figure 1.

#### 3.2.6. Primate and human studies

Rodent models are important for studying certain aspects of neurodevelopment but results cannot be directly translated to humans, as the human brain is indeed much more intricate than rodents in terms of its structure, organization, and connectivity (Boldog et al., 2018). Furthermore, difficulties in obtaining fresh human specimens make the research on human neurodevelopment much more problematic, with fewer studies available. A summary of primate and human studies on endogenous NSCs profiling by different omics techniques is reported in Table 1.

**Table 1:** Primate and human studies profiling endogenous NSCs by omics techniques.

**Reference**	**Species**	**NSC source**	**Age**	**Techniques**	**Condition**	**Major themes**
Yoon et al. (2017)	Human	human forebrain-derived organoid	post conception day 11 from a human fetus	bulk RNA sequencing	physiological	m6A mRNA modification involved in mammals’ NPC proliferation and cortical neurogenesis
Ghazale et al. (2019)	Human	human spinal cord	human 17 - and 46 years old	bulk RNA sequencing	physiological	Molecular characterization of human spinal cord NSCs niche describing pathways and gene expression
Shi et al. (2019)	Rhesus monkey	fresh cortex	prenatal stage E136; postnatal stage P76	bulk RNA sequencing	transgenic animal overexpressing human MCPH1 gene	Human MCPH1 gene involvement in neuronal differentiation and synaptic maturation delay
Yu et al. (2021)	Human	fetal sub pallium	Gestational week: 9–10–11–12	scRNA sequencing	physiological	Human sub pallium profiling and distinct interneurons gene expression characterization
Eze et al. (2021)	Human	fetal whole brain	Gestational week: 6–7–8–9–10	scRNA sequencing	physiological	Early human fetal mapping of development
Donega et al. (2022)	Human	aged SVZ	age 88 and 95	scRNA sequencing	aging	Quiescent NSCs maintenance in aged human SVZ
Donega et al. (2019)	Human	SVZ and CD271 sorted NSCs	mean age 78 ± 8	bulk RNA sequencing and proteomics	Parkinson’s disease and healthy control	CD271 sorted NSCs from PD patient transcriptome and proteome
Fernández-Muñoz et al. (2020)	Human	fetal NSCs and CSF	Gestational week: 28–42	bulk RNA sequencing	Premature infant with intraventricular hemorrhages	Novel NSCs population characterization in a premature infant with a brain hemorrhage
Chongtham et al. (2020)	Macaque monkey	SVZ	4–6 years old	bulk RNA sequencing	healthy controls and ischemic monkeys	Transient ischemia in monkey transcriptome perturbation and potential mechanism over the regeneration of brain tissue and neuroprotection
Torre-Ubieta et al. (2018)	Human	Fetal cortex (cortical plate and germinal zone)	Post conception week 15–17	ATACseq; Hi-C; bulk RNAseq	physiological	A comprehensive list of the non-coding region involved in neurogenesis highlight the distal regulatory enhancer role during brain development
Ziffra et al. (2021)	Human	Developing brain	mid gestational weeks (week 20)	scRNA-scATACseq	physiological	Comprehensive human developing brain atlas including all cortical region

Two bulk transcriptomic human studies reported new insights, focusing on the human fetal brain and the spinal cord stem cell niche (Yoon et al., 2017; Ghazale et al., 2019).

Yoon et al. (2017) performed N^6^-methyladenosine (m^6^A) mRNA sequencing on mouse E13.5 forebrain, human forebrain-derived organoids, and post-conception day 11 human fetus. This study focused on mRNA modifications by RNA methyltransferases, including Mettl3 (methyltransferaselike3-)-Mettl14 complex, Wtap (Wilms tumor 1-associated protein), Kiaa429, Rbm15 (RNA-binding motif protein 15), and its paralog (Rbm15b). In the fetal brain, enrichment of m^6^A, a conserved mRNA modification set up by the Mettl3/Mettl14 complex, was detected. m^6^A controls human cortical neurogenesis by regulating the expression of genes involved in cell cycle progression and differentiation. As m^6^A was found highly enriched in genes related to mental disorders, such as schizophrenia, it has been hypothesized that its perturbation could be a causative pathological mechanism. Depletion of m^6^A by Mettl3 knockdown showed alterations in the post-natal forebrain transcriptional profile, where a significant glutamatergic subset surprisingly persisted after cortical neurogenesis, indicating that neuronal differentiation could also occur postnatally. Indeed, m^6^A depletion correlated with the differentiation of a dysregulated glutamatergic population with low neurogenic potential and downregulation of genes involved in cell differentiation and migration (Donega et al., 2018).

The murine spinal cord stem cell niche has been described thanks to single nucleus RNA-seq, however, a deep functional and molecular characterization is necessary to unveil the potential role of spinal cord NSCs during lifespan (Habib et al., 2016). Efforts have been made in this direction, by profiling transcriptome from both human and mouse, young and adult spinal cord stem cell niches (Ghazale et al., 2019). More than one hundred transcription factors were found conserved in both mouse and human specimens. Pathway analysis revealed that smoothened/Shh and Hippo/YAP genes, known to be involved in stem cell maintenance, were enriched along with 34 members of the solute carrier family. The Tmem212 gene confirmed its relevance in the NSC spinal cord niche, specifically for the ependymal cells, in three independent studies (Rosenberg et al., 2018; Zeisel et al., 2018; Ghazale et al., 2019). These investigations are particularly relevant as they compare bulk transcriptomes of human and mouse NSCs. The highlights of those studies are reported in Table 2, with a particular focus on the similarities and differences between human and mouse transcriptomic outcomes.

**Table 2:** Similarities and differences in human and mouse bulk mRNA sequencing studies.

**Reference**	**Omics technique**	**Topic**	**NSC source mouse**	**NSC source human**	**Mouse/Human similarities**	**Exclusive to mouse**	**Exclusive to human**
Yoon et al. (2017)	bulk RNA sequencing	m^6^A mRNA landscape in neurogenesis and developmental fate transition in mouse and human brain	Primary mouse NPCs isolated from embryonic cortices at E13.5	Human forebrain derived organoid post-conception day 11 from a human fetus	Conserved regulation of cell cycle progression; shared transcripts involved in NSC, proliferation/differentiation, and neurogenesis	not reported	m^6^A methylation more prevalent; unique gene enrichment for mental disorders and neurodegenerative diseases
Ghazale et al. (2019)	bulk RNA sequencing	Molecular characterization of adult mouse and human spinal cord NSCs niche	Mouse spinal cord from 3 months old WT mice	Human spinal cord from 17 to 46 years old	Conserved expression of 1,223 genes including 120 transcription factors; epithelial organization conserved; enrichment analysis involves cilia formation, smoothened and hippo pathways, cell division, transcription, and members of the solute carrier family	Mouse ependymal zone express Slc26A3, Slc14a1, and Slc16a12; divergent organization of the ventral EZ compared to human	899 unique transcripts in the ependymal spinal cord zone

An interesting bulk study aimed at unraveling mechanisms of human brain evolution has been carried out in Rhesus monkey, which shows high similarity with Homo sapiens in terms of expression of genes involved in cell cycle regulation, apoptosis, DNA repair, and chromatin remodeling (Shi et al., 2019). The gene encoding for microcephalin-1, Mcph1, which is involved in the DNA damage response is believed to be a strong candidate that drove the evolution of the human brain size (Pulvers et al., 2015). Thanks to the generation of transgenic monkeys overexpressing human Mcph1, researchers analyzed pre- and postnatal stages of neuronal differentiation, identifying a possible explanation for the delayed neuronal corticogenesis. Indeed, Mcph1 contributes to this delay as, at the prenatal stage, transgenic monkeys showed elevated levels of transcripts related to translation, protein localization, and endoplasmic reticulum functions rather than synaptic functions, when compared to their wild-type counterparts. Furthermore, at the postnatal stage, downregulation of neuronal differentiation and maturation were observed, underscoring the role of Mcph1 in the neuronal maturation delay observed in transgenic monkeys. Altogether, these data set the basis for further studies aimed at characterizing other potential genes involved in the regulation of human CNS development (Shi et al., 2019).

The precise specification of excitatory neurons and inhibitory interneurons in the human cortex is key for the proper formation and function of neural circuits in the mammalian brain. Thanks to the enormous bioinformatics progress and to the development of specialized tools to reconstruct lineage tracing, such as RNA velocity, it has been possible to track brain development since early events, from gestational week 6–10, in different brain regions including the telencephalon, diencephalon, midbrain, hindbrain, cerebellum, ganglionic eminences, thalamus, hypothalamus and cortex (La Manno et al., 2018; Eze et al., 2021). At the earliest time points, no specific transcriptional programs across different brain regions have been observed. Mef2c, the gene encoding for the Myocyte Enhancer Factor 2C, was expressed in younger specimens (gestational week 6), and shown to play a role in early neurogenesis and synaptic formation. The analysis of samples at 10 gestational weeks showed several genes that were specifically expressed in different neuronal subsets: Nhlh1 expressed by intermediate progenitors-derived newborn neurons; Neurod6 and Bcl11b expressed by deep later neurons, and Calb2 expressed by ventrally migrating interneurons and excitatory neurons. Interestingly, the RNA velocity analysis for fate developmental mapping revealed a high number of genes associated with neurodevelopmental and psychiatric disorders that influence the differentiation program (Eze et al., 2021).

A combination of ATAC-seq and Hi-C techniques allowed to investigate chromatin accessibility and its physical interactions, and to build a comprehensive map of non-coding regulatory elements fundamental during human cortical development (Torre-Ubieta et al., 2018). Distal regulatory elements, enriched mainly in the enhancers, were found to impact neurogenesis. Transcriptome analysis confirmed and added further insights into the regulation of brain development. Specifically, germinal zone distant enhancers regulate glial differentiation genes, neuroblasts proliferation, and neurogenesis, while cortical plate distal enhancers were associated with neuron projection morphogenesis, axon guidance, and glutamate receptor activity. Moreover, the Fgfr2 enhancer revealed its fundamental role during human brain expansion. Thanks to the multimodal integration of different omics techniques, a list of enhancers regulating human brain development could be the basis for further studies that will analyze also non-coding transcripts whose genetic variations are involved in neurodevelopmental and psychiatric disorders (Torre-Ubieta et al., 2018).

Another important example of a multi-omics application is presented in the work of Ziffra et al. (2021) in which a combination and integration of transcriptome, chromatin state, and accessibility analysis create a comprehensive human developing brain atlas including prefrontal, motor, visual, somatosensory, parietal, temporal and insular cortex. Moreover, in this study, the authors describe regulatory programs during corticogenesis, which are related to risk factors of neurodevelopmental and neuropsychiatric disorders. Indeed, it was found that common schizophrenic variants were positively enriched in neuronal excitatory and inhibitory putative enhancers, confirming a previous investigation (Ripke et al., 2014). Finally, this work brings fundamental insights into the trajectory of the developing brain by associating chromatin state with transcriptome analysis, highlighting the significance of multi-omics analyses to better understand complex biological systems.

Interneurons originate in the GE in the embryonic mammalian brain and orderly migrate and settle in cortical layers before they establish cortical connections (Tremblay et al., 2016). Different types of interneurons have specific origins: OB interneurons originate in the LGE, striatal, hippocampal, and cortical interneurons originate in the MGE, while cortical interneurons originate in the CGE. This diversity has been investigated in the human fetal subpallium reporting a distinct transcriptional pattern among MGE, LGE, and CGE (Yu et al., 2021). The adult interneuron specification is already present at gestational week 9. Thanks to single-cell RNA *in situ* sequencing and analysis of the developmental trajectory, a map of each GE precursor towards a specific localized interneuron has been established. Precisely, MGE and LGE precursors induce two distinct lineages in the dorsal/ventral region respectively, thus highlighting spatial differentiation and patterning that occurs in the human fetal subpallium. A comparison of similarities and differences in the interneuron origin from initial classes of MGE, LGE, and CGE is reported in Table 3, which reports the terminal classes characterized by specific gene expression markers in mouse, primate, and human brain omics datasets recently published (Yu et al., 2021; Schmitz et al., 2022). Indeed, the work performed by Schmitz et al. (2022) dissects the initial and terminal interneurons classes across the development of mice and primates by applying RNA velocity tools and unveiling conserved initial interneurons classes of mammals.

**Table 3:** Interneurons origin comparison between mouse, primate, and human species.

	**Mouse**	**Primate**	**Human**
Initial classes	Terminal classes	Terminal classes	Terminal classes
MGE		**CRABP1/TAC3**	**CRABP1/TAC3**
	*LHX6/PVALB*	*LHX6/PVALB*	SST 57, 8%
	*LHX6/SST*	*LHX6/SST*	PVALB 54%
	*SST/CHODL*	*SST/CHODL*	ID2 14%
	*PVALB/VIPR2*	*PVALB/VIPR2*	
	*PVALB/VIPR2*	*PVALB/VIPR2*	
	*CRABP1/MAF/PVALB*	*CRABP1/MAF/PVALB*	
	*CRABP1/MAF/TH*	*CRABP1/MAF/TH*	
LGE		**TH/SCGN**	**TH/SCGN**
	*MEIS2/PAX6*	*MEIS2/PAX6*	VIP 58, 3%
	*MEIS2/PAX6*	*MEIS2/PAX6*	ID2 51.3%
	*TH/SCGN*	*TH/SCGN*	NDNF 20%
	*OXP2/CALB1*	*OXP2/CALB1*	SST 31%
	*FOXP2/4*	*FOXP2/4*	PVALP 30%
	*FOXP2/TSHZ1*	*FOXP2/TSHZ1*	
	*DRD1/NPY1R*	*DRD1/NPY1R*	
	*FOXP1/ISL1*	*FOXP1/ISL1*	
	*DRD1/NPY1R*	*DRD1/NPY1R*	
	*OXP1/PENK*	*OXP1/PENK*	
CGE	*CCK/VIP*	*CCK/VIP*	NDNF 54.9%
	*PROX1/SNCG*	*PROX1/SNCG*	VIP 21.6%
	*LAMP5/NDNF*	*LAMP5/NDNF*	SST 9.6%
	*CCK/DPY19L1*	*CCK/DPY19L1*	PVALB 6%
	*PROX1/LAMP5*	*PROX1/LAMP5*	
	*NR2F2/PAX6*	*NR2F2/PAX6*	

In the aging human brain, progenitors of the SVZ remain mostly in a quiescent state leading to neurogenesis decline. A single-cell transcriptomic study of aged subjects (88–95 years old) identified potential targets that enable the exit of NSCs from quiescence and promote their proliferation (Donega et al., 2022). The endogenous stem cell gene expression profiling in the aged is compared with published fetal datasets, showing major batch effects and issues in the correct clustering of different cell types (Zhong et al., 2018; Jäkel et al., 2019). Nowadays, studies integrating a high number of different datasets should consider applying novel tools such as SCENIC to integrate datasets in an unbiased manner which helps to avoid the batch effect (Aibar et al., 2017). Interestingly, in the dataset of aged sorted CD271 positive progenitors, the authors describe two cell clusters that were enriched in Sox10 and Rgcc gene expression, the latter being involved in cell cycle dysfunction, thus indicating that progenitors of the aged human SVZ are late OPCs (Counts and Mufson, 2017). When integrating the aged with several fetal datasets, the oligodendrocyte progenitors clustered together, nevertheless, aged OPCs were largely quiescent. Thus, the absence of mature Ols during the lifespan may be due to the persistent quiescent state of their progenitors, which is maintained *via* inhibition of the WNT signaling pathway by its antagonist Sfrp1 (Donega et al., 2022).

### 3.3. NSCs during aging

Aging is a physiological process leading to several modifications of many biological functions (Amarya et al., 2018). Neurogenesis along with the proliferation and activation rate of NSCs during aging is known to be decreased, however, the regulatory programs that govern neurogenesis in the aged human brain are still under investigation. Moreover, the literature is controversial, as divergent data were reported. Thus, whether aging induces disruption or inactivation of the NSC pool is still a matter of debate (Llorens-Bobadilla et al., 2015; Dulken et al., 2017; Basak et al., 2018; Xie et al., 2020; McCauley et al., 2021). While studies on the aging human brain have already been discussed in sub-Section “3.2.6. Primate and human studies”, we will here present studies performed in animal models.

Transcriptional dynamics in the V-SVZ of 2, 6, 18, and 22-month-old mice were analyzed by bulk RNA-seq (Apostolopoulou et al., 2017). The results show that non-monotonic changes in the gene expression program occur during aging. Many DEGs were observed at a later age. The expression of the Mash-1 genes, involved in cycle progression, is diminished in aged mice (18 months old) but is surprisingly upregulated between 18 and 22 months of age. However, in aged mice, proliferating NSCs may be more prone to cell division deregulation thus leading to neurogenesis impairment (Apostolopoulou et al., 2017).

In another study, 18 months old SVZ-NSCs showed reduced proliferation compared to 3 weeks old, confirming what was previously observed (Shi et al., 2018). Sequencing was performed using Smart-seq2, an omics technique with high accuracy and read coverage, after sorting promin-1 (Prom1)-positive NSCs (Picelli et al., 2013). Upregulated expression of the classically activated NSC markers was shown together with cell cycle-regulating genes and genes typically related to Ols and NBs. Aged cells were enriched for GO cell cycle arrest and cellular stress response. Interestingly, the transcriptional profile of the Ol lineage was different, indicating a lineage-specific aging program. The Ols lineage showed pathways related to autophagy and DNA repair mechanisms, while the NBs cluster showed pathways related to calcium and magnesium response, and reduced telomere maintenance function. Nevertheless, all three clusters showed decreased expression of cell cycle genes, strongly indicating a general decline in the proliferation rate in the aged (Shi et al., 2018).

Esophageal cancer-related gene 4 (Ecrg4) was found fundamental to aging-associated loss of NSCs proliferation (Nakatani et al., 2019). It has been demonstrated that Ecrg4-deficient mice show NSCs proliferation in the SVZ, ameliorating spatial and learning memory, with the involvement of the Foxg1 TF as a downstream mediator. However, a more complex mechanism might induce proliferation in absence of Ecrg4, which needs to be further investigated.

Overall, single-cell sequencing studies revealed that aging itself does not induce highly significant changes in the NSC niche but rather indicates a prominent quiescent state (Kalamakis et al., 2019).

Indeed, different cell clusters separate only based on DEGs that characterize quiescent and activated states. Fate mapping analyses show that the neurogenic processes from quiescent to mature neuronal and glial populations do not change during aging. Remarkably, the inflammatory state during aging strongly influences the activation of NSCs, as attenuation of inflammation in aged mice facilitates the transition from quiescence to activation. Indeed, higher expression of the Sfrp5 gene, a WNT signaling antagonist, in aged qNSCs is key, as its inhibition leads to the activation and proliferation of aged NSCs (Kalamakis et al., 2019).

Chromatin accessibility during aging has been found profoundly altered in quiescent vs. activated NSCs, accompanied by decreased accessibility of genes related to the metabolic activity (Maybury-Lewis et al., 2021). Specifically, close chromatin conformation was found for the Foxo3 gene, crucial for maintaining adult neurogenesis (Schäffner et al., 2018). This knowledge might explain the decrease in neuronal differentiation during aging but certainly need thorough elucidation.

Aging is a physiological state that greatly impacts stem cell behavior. Indeed, the aged NSC proteome in the SVZ showed impaired protein functions linked to neurological disorders, nucleic acid metabolism, cellular assembly and organization, molecular transport, and small molecule biochemistry (Wang et al., 2016). By applying Ingenuity Pathway Analysis (IPA), 39 proteins with reduced expression were associated with loss of proteostasis and neurodegenerative diseases such as AD and PD. Altogether, data confirmed the senescence phenotype of aged NSCs. Further analysis should confirm such results by studying mice older than 12 months.

Multi-omics profiling of adult and aged murine NSCs has revealed that mRNA transcription and epigenetic features remain mostly stable during aging (Lupo et al., 2018). Yet, changes at several hundred genes and regulatory elements were detected in the DNA methylation of TFs regulating the nearest genes. Dbx2, not previously described as an aging-related gene, revealed higher expression in aged NSCs and was associated with the deregulation of Sox2 and P21 gene expression, and reduced proliferation (Akizu et al., 2016). As these putative regulators of neurogenic decline changes were detected on *in vitro* cultured NSCs, only multi-omics studies on human tissue samples will provide further support to this notion.

## 4. NSCs in a perturbed environment: focus on neurodegenerative disease

Profiling NSCs in the context of neurodegenerative diseases is essential to distinguish molecular mechanisms whose physiological functions are perturbed in pathological conditions. Here, we will discuss seminal studies that have investigated NSCs, by omics technologies, in Parkinson’s disease (PD), Alzheimer’s disease (AD), brain injury, ischemic insult, and MS.

### 4.1. Parkinson’s disease

Omics technologies have been used extensively to study Parkinson’s disease to identify molecular signatures that could help improve diagnosis, treatment, and understanding of disease pathogenesis. A bulk RNA sequencing study performed in a preclinical model of PD showed important transient and prolonged transcriptomic changes in the DG of mice treated with 1-methyl-4-phenyl-1,2,3,6-tetrahydropyridine (MPTP; Bao et al., 2017). Among the transiently perturbed genes, the nuclear receptor Nr4a3 gene was upregulated and likely associated with dopaminergic innervation dysfunction upon neurotoxic treatment. The pyruvate dehydrogenase kinase Pkd4 and leukotriene receptor Gpr17 genes, respectively associated with metabolism and neuronal differentiation, showed a long-lasting upregulation. Downregulated pathways including extracellular matrix remodeling, cell junctions, proliferation, and migration were identified. Altogether, the degeneration of dopaminergic neurons in this PD model negatively impacts neurogenesis and differentiation of the DG stem cell niche as an early response (Bao et al., 2017).

Human SVZ-derived NSCs from PD patients and healthy controls (HC) have been compared using combined bulk transcriptomic and mass spectroscopy proteomic analysis, specifically investigating SVZ homogenate, and CD271 sorted NSCs (Donega et al., 2019). Whole SVZ comparison between PD and HC did not reveal any substantial differences, when comparing CD271 sorted NSCs the authors show substantial downregulation of serotonin and dopamine signaling involving Monoamine Oxidase B (MAOB) gene expression perturbation, oxidoreductases, and transcriptional activity. These results were further confirmed by proteomics where perturbed biological functions were related to metabolism and neurodegenerative disease. Moreover, NSCs in PD patients seem to be directed towards a primed phenotype as the Fgf3 gene, a quiescent stage marker, was found decreased. This phenotype might reflect an alert state, already described in other pathological conditions (Rodgers et al., 2014; Llorens-Bobadilla et al., 2015). Highlights of these data are reported in Table 1.

### 4.2. Alzheimer’s disease

Transcriptomics studies are significantly powering our understanding of the pathology of AD. The multi-omics *Atlas project—Alzheimer’s disease* is highly enriched in the novel discovery of the pathophysiology and mechanisms of AD^1^. However, most of the studies focus on mature cells such as neurons and glia, while there is missing information on NSC niches during AD development. Thus, here we report two articles from the same research group based on scRNA-seq analysis, focused on a zebrafish model of Alzheimer’s disease (AD; Cosacak et al., 2019; Bhattarai et al., 2020). The first study generated a database containing all the transcriptome information about the zebrafish developing brain, by distinguishing distinct clusters of cells in a spatially defined fashion. It demonstrated that upon amyloid β (Aβ-42) and interleukin-4 (IL-4) treatment, enhanced neurogenesis occurred driven by homogeneous sets of NBs (Cosacak et al., 2019). In the second study, the authors unveil that IL-4 treatment stimulates NSC plasticity by enhancing the BDNF growth factor released from periventricular neurons, which suppresses the serotonin effect. Moreover, BDNF binds the NGF receptor alpha (Ngfra) expressed by a subset of NSCs leading to enhanced proliferation (Bhattarai et al., 2020).

### 4.3. CNS injury

Endogenous NSCs play a relevant role in limiting inflammation and scar formation in spinal cord injury models (Patel et al., 2021). Bulk RNA sequencing of spinal cord NSCs from injured mice indicates that the NK6 Homeobox 1 (Nkx6.1) gene, known to regulate Notch signaling by controlling lineage specification, is overexpressed in the spinal stem cell niche upon injury, inducing cholinergic neuron differentiation and reducing glial formation. Nkx6.1 also boosts inflammation, mainly by activating the Notch signaling pathway. Similar effects have also been observed by overexpression of the Neurod4 TF that promotes neurogenesis and reduces glial formation in the spinal cord injury condition (Fukuoka et al., 2021).

In traumatic brain injury, an innovative therapeutic approach has been proposed to induce NSC-mediated cell replacement directly at the injury site (Chai et al., 2022). Specifically, the engineered nanofibrous structure, the aligned fibrin nanofiber hydrogel (AFG), guides the SVZ resident NSCs toward the lesion site and induces cell differentiation. Indeed, transcriptome profiling of AFG implantation in the injured mouse brain shows enhancement of the Notch and WNT signaling pathways along with increased trophins and axon guidance, suggesting that these pathways might be promising targets for the induction of lesion-specific cell replacement.

An interesting human NSC profiling was performed on cells isolated from CSF of preterm neonates undergoing intraventricular hemorrhage, a condition that occurs in 15%–40% of underweight neonates (Fernández-Muñoz et al., 2020). This condition arises in the periventricular germinal zone, where the loss of NSCs leads to dysfunctional neurogenesis and gliogenesis (Robinson, 2012). Bulk transcriptome analysis described a novel class of NSCs that closely resemble the human fetal ventral forebrain. These cells were found enriched for expression of Podxl, a glycoprotein involved in apical polarity, the IL1 receptor accessory protein Il1rap expressed in the human VZ, and other genes including Fzd5, a WNT5 receptor involved in neuronal lineage specification (Bengoa-Vergniory et al., 2017). The highlights of these results are reported in Table 1.

### 4.4. Stroke

Cerebral ischemia affects aged individuals leading, compared to young, to a worse clinical outcome. The neurodegenerative mechanisms induce neuronal cell death, leading to disability and cognitive decline (Huang et al., 2018). In this context, NSC’s ability to rescue damaged neurons remains unclear. Bulk RNA sequencing of primary murine hippocampal NSC from stroke samples demonstrates the involvement of the circular RNA circHIPK2 (Wang et al., 2020). Upon ischemic insult, the gene downstream of circHIPK2, spermine oxidase Smox, is increased and mediates the scavenging of ROS. Indeed, Smox silencing in NSCs leads to amelioration of the stroke pathology and increased neurogenesis. These results suggest that gene manipulation of NSCs for enhancement of cell replacement might represent a potential therapeutic approach to stroke.

Bulk RNA-seq of cultured murine SVZ NSCs indicates that depletion of the p75 neurotrophin receptor during cortical stroke reduces NSCs migration toward the lesion site. The p75 dysfunction might be due to cytoskeleton organization changes that impair the NSC’s migratory route acting through BDNF signaling, which increases upon CNS injury (Deshpande et al., 2022).

Ischemic insult has been also evaluated in the primate SVZ where the damage occurred spatially in the striatal and callosal subependymal layers (Chongtham et al., 2020). Bulk transcriptomic analysis of ischemic monkey NSCs revealed a major gene expression downregulation in endothelial cells, which is accompanied by oxidative stress and mitochondrial function perturbation. Upregulation of gene expression affects mainly oligodendrocytes and astrocytes by enhancement of cell adhesion, nervous system development, cell proliferation, and cell communication pathways. Intriguingly, the apelin receptor (Aplnr), a cell surface molecule promoting angiogenesis, was found upregulated upon ischemic stroke in monkeys and might contribute to post -ischemic neuroprotection and regenerative processes (Chen et al., 2015). This gene, whose variant represents a risk factor for human stroke events, should be further evaluated for an immediate therapeutic approach.

### 4.5. Multiple sclerosis

MS is a chronic, inflammatory, demyelinating, neurodegenerative disease of the central nervous system (Mey et al., 2023). The potential of NSCs in controlling progressive neuronal damage or impaired myelin regeneration has been investigated with the aim to reduce disability, maintain neuronal integrity, preventing axonal loss, and replace damaged cells (Tognatta and Miller, 2016). In this view, studies on NSC transplantation as a cell replacement strategy provide relevant clues on remyelination mechanisms. A transcriptomic analysis performed on sorted SVZ-NSCs in the cuprizone mouse model of demyelination showed that a specific subset of NSCs, enriched in the expression of Gli1, is recruited in the demyelinated site to counteract oligodendrocytes damages (Samanta et al., 2021). When cuprizone-treated mice were transplanted with Gli1-positive NSCs, they showed enriched pathways related to cellular growth and proliferation, compared to mice treated with Gli1-null NSCs. These results highlight a specific NSC population that needs further validation for the design of a potential therapeutic approach.

Single-cell transcriptomics of the brain lesions of cuprizone-treated mice revealed that re-myelination events are accompanied by the differentiation of SVZ NSCs into oligodendrocytes, which notably regulate microglia cell function (Brousse et al., 2021). While microglia cells are essential to remove myelin debris and successfully contribute to re-myelination, active inflammatory microglia might also boost demyelination (Liddelow et al., 2017; Chu et al., 2018). The interaction between NSCs and microglia is mediated by the binding of the integrin ligand milk fat globule-EGF factor 8 (Mfge8) to the microglial β3 integrin (Itgb3) receptor, which boosts microglia phagocytosis and myelin debris removal in the demyelinating context. The proposed mechanism needs further confirmation as microglial cells also expressed Mfge8.

Neurodegenerative diseases are accompanied by neuroinflammation usually arising in the surrounding tissue (Kempuraj et al., 2016). Experiments of NSC transplantation have shown that neuroinflammation influences their phenotype, differentiation capacity, and immunomodulatory function (Martino et al., 2011; Giusto et al., 2014). A recent metabolomics analysis unveiled the modulation of pro- and anti-inflammatory cytokines signaling by mouse endogenous NSCs (Drago et al., 2016). The whole (intra- and extracellular) secretome of the *in vitro* cultured undifferentiated NSCs was analyzed after exposure to pro- and anti-inflammatory cytokine cocktails. The two treatments clearly separate from each other, indicating distinct NSCs metabolomics derangement upon pro- or anti-inflammatory stimuli. Regarding the intracellular secretome, upon pro-inflammatory stimuli, the high presence of arginine, proline, phenylalanine, tyrosine, and tryptophan was found. Arginase II which is usually expressed by NSCs was found reduced upon treatment with pro-inflammatory cytokines. Similar results were found in EAE mice (as a model of MS), in which arginase II was found decreased in the SVZ during the chronic phase of the disease (Carmody et al., 2002). It is important to highlight the relevance of applying omics techniques as in this case a hypothesis-generating tool might be useful to discover novel disease biomarkers or novel therapeutic targets.

In the context of MS, increased differentiation of NSC-derived OPCs could favor cell replacement in MS lesions (Zilkha-Falb et al., 2017). Transcripts of the nuclear protein Prickle1 are strongly involved in the maturation and differentiation of OPCs toward Ols, thus suggesting Prickle1 as a potential therapeutic target in MS. Transcriptomic analysis performed on adult SVZ-derived *in vitro* differentiated cells, indicated that cell cycle suppression occurs 12 h after induction of differentiation, while neuronal and oligodendroglia gene programs are upregulated 24 h after induction of differentiation. However, *in vitro* differentiation could influence gene expression. Among the upregulated genes involved in Ols differentiation, Prickle1 essentially contributes to mature myelinated Ols development, boosting OPC-Ols in *vitro* differentiation. Prickle1 overexpression has been reported in MS brain lesions, suggesting its role as a compensatory mechanism of MS brain for Ols replacement (Han et al., 2012).

Overall, omics studies have highlighted the pathogenetic mechanisms that may contribute to MS development, and can drive the design of novel therapeutic approaches.

## 5. Conclusion

Single-cell transcriptomic analysis, enriched in direct measurement of specific cell-type transcripts in a certain tissue, has allowed a significant step forward compared to the bulk analysis. Indeed, this technology has brought insights into the understanding of the heterogeneity of the NSC niche during neurodevelopment, thus revealing the complex system required by neurogenesis to direct neuronal specification. Moreover, the development of spatial transcriptomic approaches combined with other omics techniques is providing significant insights into the spatially restricted neurodevelopmental processes. The integration of transcriptomic and chromatin profiling is essential to identify regulatory elements and pathways that control gene expression in different NSC niches.

Finally, adding proteomics and metabolomics approaches, despite the need for technological improvements, to the study of endogenous NSC biology will provide a complete picture of the complex mechanisms of brain development.

## Author contributions

VM and PP-B conceptualized and wrote the manuscript. VM performed the literature search and review, wrote the first draft, and prepared the figures. EB wrote the introduction and revised the manuscript. GM revised the manuscript. All authors contributed to the article and approved the submitted version.

## Footnotes

^1^ https://map-ad.org/publications
